# Cognitive function and nonfood-related impulsivity in post-bariatric surgery patients

**DOI:** 10.3389/fpsyg.2014.01502

**Published:** 2014-12-19

**Authors:** Ekaterini Georgiadou, Kerstin Gruner-Labitzke, Hinrich Köhler, Martina de Zwaan, Astrid Müller

**Affiliations:** ^1^Department of Psychosomatic Medicine and Psychotherapy, Hannover Medical SchoolHannover, Germany; ^2^Department of Surgery, Herzogin Elisabeth HospitalBraunschweig, Germany

**Keywords:** cognitive function, impulsivity, impulse control disorder, obesity, bariatric surgery

## Abstract

Initial evidence that cognitive function improves after bariatric surgery exists. The post-surgery increase in cognitive control might correspond with a decrease of impulsive symptoms after surgery. The present study investigated cognitive function and nonfood-related impulsivity in patients with substantial weight loss due to bariatric surgery by using a comparative cross-sectional design. Fifty post-bariatric surgery patients (postBS group) who had significant percent weight loss (*M* = 75.94, SD = 18.09) after Roux-en-Y gastric bypass (body mass index, BMI *M*_post_ = 30.54 kg/m^2^, SD_post_ = 5.14) were compared with 50 age and gender matched bariatric surgery candidates (preBS group; BMI *M*_pre_ = 48.01 kg/m^2^, SD_pre_ = 6.56). To measure cognitive function the following computer-assisted behavioral tasks were utilized: Iowa Gambling Task, Tower of Hanoi, Stroop Test, Trail Making Test-Part B, and Corsi Block Tapping Test. Impulsive symptoms and behaviors were assessed using impulsivity questionnaires and a structured interview for impulse control disorders (ICDs). No group differences were found with regard to performance-based cognitive control, self-reported impulsive symptoms, and ICDs. The results indicate that the general tendency to react impulsively does not differ between pre-surgery and post-surgery patients. The question of whether nonfood-related impulsivity in morbidly obese patients changes post-surgery should be addressed in longitudinal studies given that impulsive symptoms can be considered potential targets for pre- as well post-surgery interventions.

## INTRODUCTION

Impulsivity is considered a predisposition toward rapid, unplanned reactions to internal or external cues and by the tendency to think, plan, control, and behave insufficiently (Moeller et al., 2001). This complex construct has been linked to a broad range of psychopathology including both internalizing and externalizing symptoms (Kashyap et al., 2012; Johnson et al., 2013; Braham et al., 2014; Sharma et al., 2014). Previous research suggests at least two independent factors of impulsivity (Gray and McNaughton, 2000; Dawe and Loxton, 2004). While one factor refers to increased sensitivity to reward and approach tendencies, the other factor pertains to reduced inhibitory control and can be linked to executive dysfunctioning (Garavan et al., 2002; Hofmann et al., 2009). Given the diversity of impulsive behaviors and proposed underlying factors, the combined use of self-reports and behavioral measures has recently been recommended in order to determine impulsivity (Sharma et al., 2014). Previous findings, however, indicate a rather low or even no correlation between questionnaires and behavioral measures. This low or missing association may be explained by the fact that self-ratings and performance-based tasks assess different aspects of impulsivity (Sharma et al., 2014).

There is a growing body of research demonstrating the important role of impulsivity in overeating and obesity (body mass index, BMI 30+ kg/m^2^; Gerlach et al., 2014). For example, decreased inhibitory control toward palatable and high calorie food seems to be associated with obesity (Batterink et al., 2010; Houben et al., 2014). Food-specific impulsive responses are particularly typical for individuals with obesity and binge eating disorder (BED; Schag et al., 2013a,b). This group also shows a higher tendency toward impulsive decision making and a reduced ability to delay gratification in food-unrelated behavioral tasks (Davis et al., 2010; Müller et al., 2014a). Moreover, it appears that nonfood-related impulsive conditions such as attention deficit/hyperactivity disorder (ADHD; de Zwaan et al., 2011b; Nazar et al., 2014) or impulse control disorders (ICDs; Schmidt et al., 2012) are prevalent in individuals with obesity.

Bariatric surgery is successful in the treatment of morbid obesity resulting in long lasting weight reduction (Sjöström et al., 2007; O’Brien et al., 2013). Surgical treatment is recommended for individuals with extreme obesity (obesity grade 3, BMI ≥ 40 kg/m^2^) or for those with obesity grade 2 (BMI: 35-39.9 kg/m^2^) who suffer from chronic obesity-related somatic disorders (e.g., diabetes, hypertension, cardiovascular disease, sleep apnea, dyslipidemia; Runkel et al., 2011).

There is evidence of high psychiatric comorbidity in pre-bariatric surgery samples including BED and also other psychiatric disorders such as anxiety and affective disorders (Malik et al., 2014). Past reports demonstrated that maintenance of substantial weight loss is generally associated with a sustained improvement in psychiatric disorders (Faulconbridge et al., 2009; de Zwaan et al., 2011a; Burgmer et al., 2014). With regard to impulsive behaviors, the prevalence of BED typically decreases after surgery. Eating an objectively large amount of food is usually not possible after bariatric surgery. However, a subgroup of post-operative patients exhibits disturbed eating such as subjective binge eating, loss-of-control (LOC) over eating, picking, nibbling, etc. (de Zwaan et al., 2010; White et al., 2010; Conceição et al., 2014). Longitudinal data further suggest an increase of alcohol use two years after bariatric surgery (King et al., 2012). Questionnaire-based findings from a 2-year follow-up of patients with severe obesity treated conventionally and surgically, however, did not demonstrate significant changes in self-reported general impulsivity after treatment (Rydén et al., 2004). Taken together, valid information on post-surgery impulsive symptoms is still scarce and it remains unclear whether impulsive behaviors, other than binge eating or loss of control eating, change after surgery.

Past research indicated an association between higher BMI and impairment in cognitive function (Batterink et al., 2010; Lokken et al., 2010; Verdejo-García et al., 2010; Gunstad et al., 2012). In addition, there are assumptions in the literature concerning the contribution of poor inhibitory control to impulsive behaviors (Dawe and Loxton, 2004). Given these factors, one may expect that better cognitive control abilities would result in less impulsive behaviors in post-bariatric surgery patients compared to individuals with morbid obesity who did not undergo surgery.

To date, few studies have investigated cognitive function before and after BS. Giel et al. (2014) recently performed a longitudinal study on food cue processing. Their results demonstrated an increase in food-specific cognitive control in patients six months after surgery.

In terms of nonfood-related cognitive control, longitudinal studies suggested an improvement of neurocognitive task performance after bariatric surgery (Gunstad et al., 2011; Miller et al., 2013; Alosco et al., 2014a; Lavender et al., 2014). Participants in these studies were recruited within the Longitudinal Assessment of Bariatric Surgery (LABS) study in the US. They were presented with a computerized test battery including tasks of attention, executive function, and memory. The findings suggested improvements in cognitive function 12 weeks, 12 months, 24 months, and 36 months after surgery (Gunstad et al., 2012; Miller et al., 2013; Alosco et al., 2014a; Lavender et al., 2014).

Concurrent information on the performance in nonfood-related cognitive tasks and self-reported general impulsivity in patients with substantial weight loss due to bariatric surgery is still lacking. The present study aimed to expand previous findings investigating cognitive function and food-unspecific impulsivity in a group of post-bariatric surgery patients (postBS group) by using a combination of behavioral tasks, self-ratings and clinical interview. In addition to tasks focusing on attention, mental flexibility, and response inhibition, we decided to apply tasks on decision-making and problem solving. Poor performance in such tasks is considered to be associated with disadvantageous decisions and an impulsive responding style (Bechara and Martin, 2004; Goudriaan et al., 2005; Davis et al., 2010; Müller et al., 2014a). To the best of our knowledge, differences in decision-making and problem solving before and after surgery have not been examined in prior studies. Therefore, we assumed that the inclusion of these tests would add to the literature.

The postBS group was compared with an age and gender matched group of pre-bariatric surgery patients (preBS group). Based on previous research on cognitive functioning in bariatric surgery samples our hypotheses were twofold. First, we expected to find a better performance in neuropsychological tasks in the postBS group compared to the preBS group. Second, we hypothesized that the postBS group would have less impulsive symptoms given the aforementioned expected better cognitive abilities after bariatric surgery. In addition, we aimed to examine the relationship between the measures that were thought to tap different aspects of impulsivity, in particular performance-based tasks, questionnaires, and clinical interview. Based on earlier findings we expected a rather weak correspondence.

## MATERIALS AND METHODS

### PARTICIPANTS AND STUDY DESIGN

The postBS group consisted of 50 patients who had received a Roux-en-Y gastric bypass. They were recruited at the Department of Surgery of the Herzogin Elisabeth Hospital Braunschweig. This group was matched by age and gender with 50 pre-bariatric surgery patients (BMI ≥ 30 kg/m^2^) from another study that had examined the role of somatic comorbidity, executive functions, and temperament in obesity (Kiunke et al., 2013; Müller et al., 2014a,b). The preBS group included bariatric surgery candidates who were scheduled for a Roux-en-Y gastric bypass operation. They were recruited within a preoperative psychiatric evaluation at the Hannover Medical School (*N* = 19) or the University Hospital Erlangen (*N* = 31) and had completed the same assessments as the postBS group plus two additional cognitive tasks (Kiunke et al., 2013). The order of measurements was the same across groups but the assessments lasted longer in the preBS group (1.5 h vs. 2 h) due to the additional tasks that were administered at the end of the cognitive assessment. The preoperative psychiatric evaluation and the assessments for this study were conducted by different independent assessors. All patients were assured that information provided for the present research study would not influence their candidacy for surgery.

Inclusion criteria for both groups were age between 18 and 65 years and sufficient German language skills. Exclusion criteria were any neurological disorder, current substance abuse, psychosis, suicidal ideations, sensory impairments, mental retardation, and any developmental or learning disorder.

Data were obtained between May 2011 and May 2013. Participation in the study was completely voluntary and written informed consent was obtained from all participants. The protocol was approved by the Institutional Ethics Committee of the Hannover Medical School.

### ASSESSMENT

#### Cognitive function

The tendency toward food-unspecific impulsive, disadvantageous decisions was measured by using a modified computerized version of the Iowa Gambling Task (IGT; Bechara et al., 1999). Over a task of 100 trials, patients were instructed to choose cards from four decks (A, B, C, and D). All cards contained either monetary profits or losses. The participants started with an amount of 2000€ and they were instructed that the goal of the task was to win as much money as possible until the test stopped. Decks A and B were considered as “disadvantageous” given that they yielded high monetary profit but also higher losses, decks C and D were viewed as “advantageous” as they yielded lower monetary profit but also lower losses. The IGT performance was measured by net scores that were calculated by subtracting the total number of disadvantageous cards from the total number of advantageous cards [(C + D) – (A + B)]. Lower scores (more card selections in decks A and B) indicate poorer learning ability and/or an increased tendency toward impulsive choices.

A computerized version of the Tower of Hanoi (ToH) was used to measure problem solving abilities (Welsh and Huizinga, 2005). The patients were asked to transfer four disks from a start rod to a goal rod in the fewest number of moves as possible while following specific rules. This complex problem-solving task requires working memory, planning, and inhibition. Poor performance is considered to be linked to an impulsive response style. The ratio between the number of ideal disk moves and the number of actually needed disk moves (effectivity) was the dependent variable.

Cognitive control including selective attention and response inhibition was assessed using a computerized version of the Stroop Test (MacLeod, 1991). The participants had to touch the correct button on the touch screen and could not give the answer verbally. The outcome variable was the number of correct answers in the interference condition, were the participants were asked to correctly name the color a word was written in instead the actual word itself. Higher scores indicate better cognitive control.

The Trail Making Test-Part B (TMT-B; Reitan, 1992) was used to measure mental flexibility and the ability to switch attention. Participants were asked to tap numbers (1 through 9) and digits (A to I) in an alternating sequence as quickly and as accurately as possible. Time to complete this task in seconds was used as outcome variable. Lower scores indicate better cognitive abilities.

The Corsi Block Tapping Test (Corsi) was administered to assess the capacity of visuospatial short-term memory (Berch et al., 1998; Kessels et al., 2000). Participants had to reproduce several sequences of block tappings displayed only once by the computer. When the sequence was correctly copied, the number of cubes which had to be touched was increased step-wise. The outcome variable of this task was the total number of correct answers.

#### Self-reported impulsivity

The Behavioral Activation System (BAS) scale of the German version (Strobel et al., 2001) of the Behavioral Activation System/Behavioral Inhibition System scales (BIS/BAS; Carver and White, 1994) was used to assess dispositional approach tendencies and reward sensitivity (Müller et al., 2014b). The BAS-scale consists of 13 items (e.g., “I go out of my way to get things I want”). Cronbach’s α in the present study sample was 0.68.

Data on the German version of the Conners Adult ADHD Rating Short-Scale-self-report (CAARS; Christiansen et al., 2013) were available on 41 pre-bariatric and 47 post-bariatric surgery patients. The CAARS subscale ‘Impulsivity’ was used to measure food-unrelated impulsivity. This subscale includes five items (e.g., “I interrupt others when talking”). Cronbach’s α in the present study sample was 0.80.

#### Interview-based impulsivity

Impulse control disorders were measured using the ICD module of the research version of the Structured Clinical Interview for DSM-IV (First et al., 2002). The interview includes sections for intermittent explosive disorder, non-paraphilic compulsive sexual behavior, kleptomania, trichotillomania, skin picking, pathological gambling, pathological internet use, and compulsive buying. As some patients may develop excessive exercising after bariatric surgery, we added a module to assess excessive exercising. According to the interview, a current diagnosis refers to the occurrence of an ICD within past 6 months. Assessment was carried out by four doctoral level students who were trained in a standardized way beginning with observations of interviews followed by a series of interviews which were reviewed by the last author. All assessors were regularly supervised by the last author.

#### Descriptive variables

To describe the sample, information on weight, height, and obesity-related somatic comorbidity (in particular: hypertension, diabetes, sleep apnea, dyslipidemia, pain disorder) was taken from patient charts. Age, nationality, and information on school education were self-reported.

The presence or absence of BED was assessed by interview using the BED module of the German version of the Eating Disorder Examination (Hilbert et al., 2004). Patients were diagnosed with BED if they had at least two binge eating episodes (eating an objectively large amount of food with a sense of loss of control) per week without recurrent use of inappropriate compensatory behavior over a period of six months according to the DSM-IV-TR appendix criteria for BED (American Psychiatric Association [APA], 2000). In addition, the EDE-Questionnaire (EDE-Q; Hilbert and Tuschen-Caffier, 2006) item 14 (“Over the past four weeks on how many times did you have a sense of having lost control over your eating?”) was used to assess the presence of LOC eating over the last 4 weeks.

### DATA ANALYSIS

Percent excess weight loss (%EWL) was defined based on BMI changes by using the following formula: (preoperative BMI-postoperative BMI)/25 × 100.

Statistical analyzes were performed using IBM SPSS Statistics version 22. Chi-squared tests were used to compare the two groups with regard to categorical variables. To calculate differences between the groups in continuous variables, we performed independent sample *t*-tests or Mann–Whitney’s-*U* tests when the variables were not normally distributed. Two-tailed Spearman’s rank-order correlations were run to determine the relationship between the different measures. The significance level for all tests was set at α = 0.05.

## RESULTS

### DESCRIPTIVE CHARACTERISTICS

The postBS group reported an average time after surgery of 14.44 months (SD = 3.59, range: 9–28) and a mean %EWL of 75.94% (SD = 18.09, range: 34.84–113.64). The current BMI of the pre-surgery group did not differ significantly from the pre-surgery BMI of the post-surgery group (*M*_pre_ = 48.01 kg/m^2^, SD_pre_ = 6.56 vs. *M*_post_ = 49.52 kg/m^2^, SD_post_ = 5.79, *t*_(98)_ = -1.22, *p* = 0.224). Both groups were matched by age and gender; thus, no group differences were found on these variables. The mean age of the preBS group was 42.04 years (SD = 11.17) and the postBS group was 42.30 years (SD = 10.64). Each group consisted of 43 women (86%) and 7 men (14%).

As can be seen in **Table 1**, the groups differed in terms of BMI categories with the preBS group showing a significantly higher mean BMI compared to the postBS group (*M*_pre_ = 48.01 kg/m^2^, SD_pre_ = 6.56 vs. *M*_post_ = 30.54 kg/m^2^, SD_post_ = 5.14, *t*_(98)_ = 14.82, *p* < 0.001). Patients in the preBS group were more likely to have a comorbid somatic condition.

**Table 1:** Descriptive variables.

	preBS *N* = 50	postBS *N* = 50	Group comparison	
	*N (%)*	*N (%)*	* χ^2^*	*p*
**BMI categories**
Normal Weight^a^	–	5 (10.0)	77.08	< 0.001
Overweight^b^	–	22 (44.0)		
Obesity Grade 1^c^	–	13 (26.0)		
Obesity Grade 2^d^	5 (10.0)	7 (14.0)		
Obesity Grade 3^e^	45 (90.0)	3 (6.0)		
Any somatic disorder	33 (66.0)	9 (18.0)	23.64	<0.001
Binge Eating Disorder (BED)^f^	16 (32.0)	–	19.05	<0.001
Loss of Control Eating^g^	15 (34.9)	4 (8.3)	12.70	0.002
**Nationality**
German	45 (90.0)	46 (92.0)		
Turkish	3 (6.0)	2 (4.0)	3.68	0.451
Russian	1 (2.0)	2 (4.0)		
Polish	1 (2.9)	–		
**School years**
<9	17 (34.0)	16(32.0)	5.68	0.128
10	23 (46.0)	27 (54.0)		
11–13	5 (10.0)	7 (14.0)		
>13	5 (10.0)	0 (0.0)		

*^a^BMI 18–24.9 kg/m^2^, ^b^BMI 25–29.9 kg/m^2^, ^c^BMI 30–34.9 kg/m^2^, ^d^BMI 35–39.9 kg/m^2^, ^e^BMI ≥ 40 kg/m^2^; ^f^interview-based diagnosis according to the DSM-IV-TR appendix criteria for binge eating disorder, ^g^at least four episodes during the last 28 days according to the Eating Disorder Examination-Questionnaire, information available from 43 preBS and 48 postBS patients.*

While about one third of the preBS group was diagnosed with BED, none of the patients in the postBS group met the criteria for BED. Also, at least one LOC eating episode per week during the last month occurred more often in the pre-operative group. No group differences were found in nationality or school education (see **Table 1**).

### COGNITIVE FUNCTION AND NONFOOD-RELATED IMPULSIVITY

**Table 2** demonstrates a lack of significant group differences with regard to cognitive function, self-reported impulsivity or interview-based diagnoses of any ICD. The group difference in the prevalence of any current ICD at assessment did not reach significance (preBS 10.0% vs. postBS 20.0%, *X*^2^_(1)_ = 2.08, one-tailed Fisher’s exact test *p* = 0.122, respectively).

**Table 2:** Group comparison on cognitive function, self-reported, and interview-based nonfood-related impulsivity.

	preBS group	postBS group	Group comparison	
	*M* (SD)	*M* (SD)		*p*
**Cognitive function**
Iowa Gambling Task	-8.98 (13.24)	-5.96 (14.16)	*t*_(97)_ = -1.096	0.276
Tower of Hanoi	0.52 (0.24)	0.48 (0.24)	*t_(97)_* = 0.805	0.423
Stroop Test	18.66 (2.68)	18.34 (2.10)	*t*_(98)_ = 0.664	0.508
Trail Making Test, Part B	7768.71 (2675.90)	7877.07 (2316.25)	*t*_(98)_ = -0.216	0.829
Corsi Block Tapping Test	6.41 (2.81)	5.77 (1.93)	*U* = 1059.00^a^	0.394
**Self-reported impulsivity**
BAS	2.97 (0.31)	3.07 (0.30)	*t_(98)_* = -1.719	0.089
CAARS-Impulsivity	3.76 (2.60)	4.19 (2.88)	*t*_(86)_ = -0.741	0.461
**Impulsive behaviors (interview)**
Number of current impulse control disorders	0.12 (0.39)	0.33 (0.75)	*U* = 1091.50^b^	0.134

*BS, bariatric surgery; BAS, Behavioral Activation System scale; CAARS, Conners Adult ADHD Rating Short-Scale-self-report.*

*^a,b^Mann–Whitney’s-*U* test was used because of non-normal distribution. Mean ranks of preBs and postBS: ^a^51.39 and 46.56, respectively, and ^b^47.33 and 52.72, respectively.*

### CORRELATION BETWEEN MEASURES

**Table 3** displays the two-tailed Spearman’s rank-order correlations between variables.

**Table 3:** Two-tailed Spearman’s rank-order correlations between variables.

	ToH	Stroop	TMT-B	Corsi	BAS	CAARS-Imp	ICD
IGT	0.200* (*N* = 99)	-0.009 (*N* = 99)	-0.015 (*N* = 99)	0.022 (*N* = 97)	0.186 (*N* = 99)	-0.083 (*N* = 87	-0.115 (*N* = 98)
ToH		0.119 (*N* = 99)	-0.241* (*N* = 99)	-0.024 (*N* = 97)	0.080 (*N* = 99)	-0.027 (*N* = 87)	0.061 (*N* = 98)
Stroop			-0.169 (*N* = 100)	0.250* (*N* = 97)	-0.041 (*N* = 100)	0.036 (*N* = 88)	0.048 (*N* = 99)
TMT-B				-0.360** (*N* = 97)	-0.128 (*N* = 100)	0.031 (*N* = 88)	-0.192 (*N* = 99)
Corsi					0.024 (*N* = 97)	-0.030 (*N* = 85)	-0.039 (*N* = 96)
BAS						0.098 (*N* = 88)	-0.002 (*N* = 99)
CAARS-Imp							0.306** (*N* = 87)

IGT, Iowa Gambling Task; ToH, Tower of Hanoi; TMT-B, Trail Making Test-B; Corsi, Corsi Block Tapping Test; BAS, Behavioral Activation System scale; CAARS, Conners Adult ADHD Rating Short-Scale-self-report, ICD, number of impulse control disorders. *p < 0.05; **p < 0.01.

More correct answers in the Corsi Block Tapping Test were related to shorter completion time in the TMT-B and to better performance in the Stroop Test. Outcomes in the ToH and the IGT were positively correlated. Moreover, we found a significant relationship between scores on the CAARS subscale ‘Impulsivity’ and the number of impulse control disorders.

## DISCUSSION

The main result of the present study is that we did not find significant differences between patients after significant weight loss due to bariatric surgery and patients seeking surgical treatment for obesity with regard to cognitive function or nonfood-related impulsivity.

In contrast to our first hypothesis, the postBS group did not show a better performance in neuropsychological tasks than the preBS group. Given initial evidence from a few longitudinal studies demonstrating increased cognitive control after surgery (Gunstad et al., 2011; Miller et al., 2013; Alosco et al., 2014a,b; Lavender et al., 2014) we expected a better test performance in the post-surgery group than we observed. The respective prior studies found post-surgery improvement amongst others in domains that were also assessed in our study, i.e., verbal interference (adopted version of the Stroop task), and switching of attention (adopted version of the TMT-B; Miller et al., 2013; Alosco et al., 2014a,b). Past studies had not used tasks on decision-making or problem solving. Therefore, we have applied the IGT and the ToH. However, the groups did not differ in these tasks either.

In addition to variances in measurement, another important difference between our study and previous studies is the fact that we compared two different cross-sectional samples, whereas earlier studies used longitudinal designs. The intra-individual variability in test performance in longitudinal studies is probably lower than possible inter-individual differences between cross-sectional samples that may have contributed to the lack of group differences in our study. Nonetheless, the present age and gender matched postBS and preBS groups did not differ in pre-surgery BMI and seemed quite typical for post- and pre-surgery samples in terms of BMI, somatic comorbidity, and the absence/presence of BED and LOC eating (Mühlhans et al., 2009; O’Brien et al., 2013; Burgmer et al., 2014; Mitchell et al., 2014). Therefore, it cannot be completely ruled out that our results indicate a real absence of pre- and post-surgery group differences in cognitive control and impulsivity symptoms.

To date, information on general impulsivity pre and post-surgery remains scarce. In the present sample, impulsive symptoms as measured by questionnaires or diagnosed by clinical interview did not vary between pre and post-surgery patients. These results support the findings of Rydén et al. (2004) who did not find changes in general impulsivity after weight loss due to bariatric surgery or conventional treatment.

An additional aim of the present study was to examine the correspondence between different cognitive and impulsivity measures, which we expected to be weak or absent based on the existing literature (Sharma et al., 2014). Indeed, we found very few significant correlations. A positive relationship was identified between the number of diagnosed ICDs and the subscale ‘Impulsivity’ of the CAARS which seems plausible. Also, some of the outcomes in the cognitive tests were significantly related.

Our results should be interpreted in the context of certain limitations. The most noteworthy of these limitations is our cross-sectional comparison of two independent patient groups, which may restrict the conclusions that can be drawn from our results. Additional studies addressing cognitive function and different facets of impulsivity after bariatric surgery should use adequately powered longitudinal designs with long-term observations. Another shortcoming of this study is that we relied on the BAS scale and the CAARS ‘Impulsivity’ subscale to measure self-reported impulsivity. The CAARS subscale might not have reflected the whole impulsivity spectrum. According to Carver and White (1994) the BAS scale measures temperamental reactivity toward potential non-punishment or rewarding outcomes. Because this dispositional impulsivity is considered to be rather stable over time, the questionnaire is probably not very sensitive to change and that may explain the lack of group differences. Also, potential post-surgery impulsive behaviors other than ICDs could have been of interest but were not assessed in the present study, e.g., substance abuse or non-suicidal self-injury.

Overall, the present findings confirm earlier results with regard to significant weight loss, and include decreased somatic comorbidity, and less BED in post-bariatric surgery patients compared to patients with morbid obesity who did not undergo surgical treatment. With regard to cognitive function and nonfood-related impulsive symptoms, however, our results indicate a lack of group differences.

## Conflict of Interest Statement

The authors declare that the research was conducted in the absence of any commercial or financial relationships that could be construed as a potential conflict of interest.
